# “I want to be healthy and move on”: A qualitative study of barriers and facilitators to antiretroviral treatment adherence among young adult survivors with perinatal HIV in Thailand

**DOI:** 10.1371/journal.pone.0305918

**Published:** 2024-07-16

**Authors:** Linda Aurpibul, Arunrat Tangmunkongvorakul, Chanidapa Detsakunathiwatchara, Angkana Srita, Supunnee Masurin, Patcharaporn Meeart, Walailak Chueakong

**Affiliations:** Research Institute for Health Sciences, Chiang Mai University, Chiang Mai, Thailand; University of Lincoln College of Science, UNITED KINGDOM

## Abstract

We know that HIV treatment outcome depends on antiretroviral treatment (ART) adherence. Young adults with perinatal HIV (YPHIV) who survived have endured various adherence challenges in their adolescent years. While some of them could maintain perfect adherence with sustainable virologic suppression, many experienced one or more episodes of virologic failure. We explored factors affecting ART adherence from real-life experiences of YPHIV. A qualitative study was conducted between June and November 2022. Twenty YPHIV aged 21–29 years with a history of virologic failure and resumed virologic suppression during adolescent years were invited to share their experiences through individual in-depth interviews. Audio records were transcribed verbatim and analyzed using deductive thematic analysis. We divided excerpts into two themes: barriers and facilitators to ART adherence. The socio-ecological model was used to frame subthemes at personal, societal, and healthcare system levels. Most barriers to adherence were concentrated at the personal level, including work/study-related conditions, personal entertainment, medication issues, mental health problems, thought, and belief. At the societal level, social activities and fear of HIV disclosure were frequently mentioned as barriers. Medical care cost was the only identified barrier at the healthcare system level. The facilitators to adherence at the personal level included perceiving health deterioration, being afraid of hospitalization and medical procedures, and wishing to be healthy and move on. At the same time, perceived family support and determination to complete family without HIV transmission were identified as facilitators at the societal level. Service behaviors of healthcare providers were mentioned as facilitators to adherence at the healthcare system level. From this study, most factors associated with non-adherence in adolescents were at the personal level, and the fear of HIV disclosure was critical at the societal level. The key facilitator to adherence was the determination to be healthy and have a promising future. Our findings reinforce the importance of establishing youth-friendly services in the existing HIV care setting. More time allocation for tailored individual counseling, using other novel approaches like mHealth, online media, and involvement of social support from different sectors might be beneficial to maximize adherence self-efficacy during the transitional period of YPHIV.

## Introduction

Today with the effective antiretroviral treatment (ART), people living with HIV are healthy and live near normal life expectancy [1]. However, a greater than 95% adherence to ART is necessary for durable virologic suppression [2]. ART adherence is defined as the ability to develop and follow a plan of behavioral and attitudinal changes that ultimately serves to empower them to manage a given health condition [3]. It could not be perfect in many life circumstances. According to a meta-analysis which included studies from all age groups worldwide, frequent reported barriers to adherence included forgetting, being away from home, and a change in daily routine [4]. An Indonesian study among transgender women living with HIV also mentioned worked-related tiredness, financial problems, and seeing that friends with HIV who did not receive ART remained healthy as barriers ART adherence [5]. A Laos study reported being busy, being forgetful, illicit drug use, certain lifestyle and education level were associated with non-adherence [6], while a Thai study reported mental health symptoms, access to care, HIV disclosure and family communication as factors related to adherence in PLHIV [7].

Unlike adults who are self-responsible for their medication immediately following the treatment initiation, adherence of those with perinatal HIV infection depends on the caregivers during childhood. Life becomes complicated while transitioning from childhood through adolescent years towards adulthood. Besides their struggle with healthcare transition [8], the physical, psychosocial, and life situation changes could affect ART adherence during adolescent years [9, 10], either with or without change in healthcare access. The rates of ART adherence among adolescents from different regions varied between 53–84% [10]. A longitudinal study in the US reported that the prevalence of nonadherence and unsuppressed viral load significantly increased with age from pre-adolescence to late adolescence [11]. However, not all could make it through the vulnerable period; previous reports revealed an association between poor adherence, advanced HIV diseases and death in adolescents with HIV [12–14].

Globally, of the estimated 600,000 AIDS-related death in 2022, approximately 16% were children/adolescents aged under 20 [15]. In Thailand, as of 2022, there were around 560,000 PLHIV, 11,000 people who died of AIDS, and 1,700 children under 14 were receiving HIV care [16]. The National HIV/AIDS Treatment program has offered HIV treatment free-of-charge for children in Thailand since 2007 [17]. Many pediatric patients started ART in their school ages now became young adults with perinatal HIV (YPHIV) with a long history of ART. We observed episodes with increased HIV viral load, presumed virologic failure, and subsequently resumed virologic suppression in their medical records. As ART is a lifelong treatment and ART adherence is a dynamic process, we perceived adherence challenges during adolescence and young adult life could be manageable and preventable. Factors related to adherence specific to this vulnerable population, which might differ from one setting to another, should be addressed. In this study, we explored barriers and facilitators to ART adherence in YPHIV who survived through the tough time in their adolescent years and were currently virologic suppressed. Learning from their experiences would allow more understanding of the context and enable healthcare providers and those in supportive roles at different levels to offer more comprehensive services or improve existing programs for better health outcomes, including fewer morbidities and mortality during this transitional period of life and later on.

## Materials and methods

### Study setting

The study was conducted at the Family clinic, Research Institute for Health Sciences, Chiang Mai University. Chiang Mai is a metropolitan city in northern Thailand where the prevalence of HIV/AIDS was high since 1995 [18]. When ART was available, many children with perinatal HIV and severe immunosuppression were initiated on ART. When they grew up and HIV treatment program was widely implemented, they were transitioned from pediatric clinics to HIV clinics for adult patients during adolescent years at different ages depending on each clinic policy. Many YPHIV remained in the city and could be contacted through online and offline social networking. We used a qualitative approach to provide comprehensive understanding of factors affecting ART adherence during their adolescent years. The Consolidated criteria for reporting qualitative research (COREQ) was adopted for planning and conducting the study [19].

### Study population

Purposive sampling strategy was used to recruit YPHIV. We asked outreach workers who were familiar with YPHIV in communities, and healthcare providers who provided HIV care to YPHIV in clinics to offer the study information to potential participants by either face-to-face meeting or phone call. The inclusion criteria were 1) aged 20–30 years, 2) living with perinatal HIV, 3) have experienced virologic non-suppression during their adolescent years and could resume their virologic suppression later, and 4) willing to join the study and share their experiences. After hearing from outreach workers or healthcare providers, prospective participants who expressed their interest to participate were invited to the study site to learn about the study purpose. Written informed consent was obtained from those who agreed to join. The targeted number of participants of 20–25 was estimated to reach data saturation [19].

### Data collection

We collected data from individual in-depth interviews (IDI) with each participant. The interview took place in an undisturbed room in the Family Clinic within the clinical research unit. Demographic information included sex, age, duration on ART, highest level of education, marital and job status were obtained. All authors (one MD, one PhD, 3 registered nurses, 2 clinical research assistants with Bachelor’s degree) conducted the interview. All are female and were trained on qualitative interviews and have clinical experiences working with this population. None had personal relationships with or being current healthcare providers for the study participants. Participants were informed about the study aims during the consent process. During each interview session, only an interviewer and a participant were present. Each IDI started with a brief self-introduction by the interviewer. We used a study interview guide developed by the study team based on a thorough literature review to facilitate conversation around factors influencing ART adherence during adolescent years of young adult survivors. No repeated interview was carried out. All interviews were audio recorded with participants’ permission. Each interviewer wrote a field note after the interview session ended. We did not return the transcripts to participants. The data saturation was discussed in the research meeting.

### Data analysis

Qualitative data from IDI audio files were transcribed into verbatim (word by word transcripts). We used Dedoose Software (Dedoose Version 9.0.90, a web application for managing, analyzing, and presenting qualitative and mixed method research data (2023). Los Angeles, CA: Sociocultural Research Consultants, LLC www.dedoose.com.) which is a user-friendly web-based program for data analysis. The transcripts were read line-by-line by two research team members then shared and discussed between the interviewer and another research team member. Deductive-focused analysis was applied. A code book was developed and used in coding all transcripts by two independent coders. A theme matrix was constructed based on the coded data. We divided excerpts under two themes: barriers, and facilitators to ART adherence in the adolescent period. The socio-ecological model was used as a framework to categorize excerpts into three levels (subthemes: personal, societal, and healthcare system). The model would allow an understanding of factors at each level and guide the future development of comprehensive intervention approaches. All interview transcripts analysis were in Thai. Selected excerpts were translated into English during manuscript preparation to support the results in each theme.

### Ethics consideration

The study involving humans was approved by the Human Experimentation Committee, Office of Research Ethics of the Research Institute for Health Sciences, Chiang Mai University (certificate of approval no. 21/2022). The study was conducted in accordance with local legislation (i.e. Personal Data Protection Act) and institutional requirements. The participants provided their written informed consent to participate in the study and were confirmed about their right to stop the conversation or ask the interviewer to stop recording at any time.

## Results

### Demographics of study participants

From June to November 2022, we recruited 20 YPHIV and all of them were enrolled; 8 (40%) were female. The median age of participants were 25 years (interquartile range, IQR 22–28), and duration on ART were 17 years (IQR 14–19). Their characteristics are shown in Table 1.

**Table 1:** Demographic of study participants (n = 20).

Characteristics	Total	Female	Male
	n = 20	n = 8	n = 12
Age (years)	25 (22–28)	24 (21–26)	26 (24–28)
Duration on antiretroviral treatment (years)	17 (14–19)	17 (15–19)	17 (14–20)
Highest education			
Primary school	2 (10%)	0	2 (17%)
Secondary school	5 (25%)	3 (38%)	2 (17%)
High school	3 (15%)	1 (12%)	2 (17%)
Vocational school	10 (50%)	4 (50%)	6 (50%)
Relationship			
Single/ no relationship	14 (60%)	7 (88%)	7 (58%)
Coupled/ Separated	6 (30%)	1 (12%)	5 (42%)
Job status			
Studying	1 (5%)	1 (12%)	0
Working	18 (90%)	7 (88%)	11 (92%)
Unemployed	1 (5%)	0	1 (8%)

Number (%) or median (interquartile range)

The duration of interview ranged from 50–90 minutes. From the interviews, we found that factors affecting adherence in adolescent years of YPHIV were concentrated on the personal level, and more barriers were mentioned than facilitators. The coded excerpts were organized into categories under each subtheme and theme (Table 2).

**Table 2:** Themes, subthemes, and categories of factors affecting adherence in adolescent years.

Themes	Subthemes	Categories
**Barriers**	Personal level	Work/study-related
		Personal entertainment
		Medication issues
		Mental health, thought and belief
	Societal level	Social activities
		Unexpected life situation
		Fear of HIV disclosure
	Healthcare system level	Medical care cost
**Facilitators**	Personal	Perceived health deterioration
		Being afraid of hospitalization and medical procedures
		Wish to be healthy and move on
	Societal level	Perceived family support
		Desire to complete family without HIV transmission
		Perceiving responsibility for their children
** **	Healthcare system	Services behaviors of healthcare providers

### Barriers at the personal level

**Work/study-related.** YPHIV in our study started working very young after finishing primary or secondary school. According to the interviews, they did not often forget their medication time but missed the opportunity to take it as planned. Work- and study-related reasons prohibited them from adhering to ART, especially when starting a new job or working under circumstances. A man shared the situation in a restaurant where he was working to explain how he missed his medicine.

*“I worked in a fast-food restaurant*. *I spent most of the time frying chicken. I had to do everything in a limited time, to be ready for sell. It was a continuing process, picking them out, cooking them, carrying them to the front, and other things to be done. I could not…just left it there. To take med, I would need to clean my hands. It would waste time and my boss would be upset if things were not ready. So, I missed my pills.” (048, male 29 years)*

A young man claimed he always forgot to bring the pill to work and only recognized it when the cell phone rang in his pocket. Another guy used to work in a workplace with strict biosafety regulations and was not allowed to carry any items.

“*I was busy working in a barbeque restaurant*. *My working hours were in the evening*, *and sometimes I forgot to bring my med with me*.*” (044*, *male)*

“*I worked in a food processing factory*. *There was a sensor at the entrance*. *We needed to leave our personal items in the lockers provided outside during the working hours*. *When I worked on 12-hour shift or longer*, *I missed my dose*.*" (049*, *male 25 years)*

Some reported working at different hours or being too tired after work and unable to stay awake until their regular dosing time as causes of non-adherence.

"*I worked as a security guard on 12-hour shift*. *I was sleepless*. *My timetable changed*. *I took my pills at different time each day*, *and some days I absolutely forgot about them*.*" (042*, *female 26 years)*

“*I was so anxious with my work*. *When arriving at home, I was too tired to do anything" (057, male 28 years)*

Another woman mentioned she was overloaded with working and studying assignments, which prevented her from adhering to the planned dosing time.

“I *totally forgot about taking med during that time*, *when I had a ton of homework to do*. *I also worked part-time while studying in the vocational school*. *Both working and studying took all my time and concentration*.*” (054*, *female 21 years)*

#### Personal entertainment

Being on their own with entertaining activities was also mentioned as a reason for non-adherence. A man did not continue his education after finishing the secondary school. His biological parents worked fulltime while he spent his days at home alone.

“*When I quit school*, *I stayed at home playing games every day*. *My days were like… drinking [alcohol]*, *playing games*, *drinking [alcohol]*, *and playing more games after games*. *That was all in my life during that moment*. *I knew it was my dosing time when the phone alarmed*, *but it would waste my game time*, *I just ignored it and continued playing*.*” (050*, *male 25 years)*

#### Medication issues

In the adolescent years, YPHIV needed to manage their dosing. A woman recalled her childhood experience with her grandmother’s support and the change when she had to be responsible for her own life.

“*I did not take it*, *the one with bad smell*. *When I was a kid*, *my grand had a glass of milk for me to take with the pill*. *When I was on my own*, *I did not have any beverages*, *and it was like…nauseating to me*.*” (046 Female 23 years)*

Many YPHIV have switched regimens in their adolescent years following the national guideline from a twice to a once-daily regimen composed of efavirenz. The neuropsychiatric side effects were more than mild, interfered with their daily activities, and led to their self-interruption of ART.

“*The drug made me dizzy*. *I could not work or sleep and had a bad dream*. *I told the doctor*, *and he said those symptoms would go away after 1 or 2 months*, *but they weren’t*. *I stopped it myself as I was so suffered*.*” (056*, *male 27 years)*

“*The pill had a bad smell*. *I could not take it*. *I told the doctor*, *and she prescribed another drug for me*. *The new one that caused dizziness*. *I was sleepy and blurred*. *They were the side effect*, *I thought*.*” (060*, *female 28 years)*

#### Mental health, thought and belief

YPHIV mentioned mental health symptoms like depression as a cause of non-adherence in adolescents. One recalled his feeling down around the age of 18–19. He had many questions about himself, and he was not satisfied with the answers from his primary provider at that time.

“*When I was a senior in high school*, *I had a lot of questions about myself*, *my disease*, *my med*, *and other things*. *I kept asking the doctor and I felt down*. *It was like my life was down*, *and I did not want to do anything*. *After finishing high school*, *I got a job at a superstore*, *and I stopped taking medication*.*” (056*, *male 27 years)*

The same informant stated that his curiosity continued to his late adolescent years. He had another non-adherence period when he was depressed, confused, and unable to proceed his own life.

“*I was depressed*, *severely depressed*. *I wanted to die*. *Back then*, *I had a girlfriend*. *We were together for a year*, *I thought she was the one I wanted to live with*. *But … I kept wondering*, *how could we have sex*, *if we would be able to have kids*, *how would I tell her about my… I was so confused at that time*. *I decided to leave everything*, *stay alone*, *and did not show up at the clinic again*.*” (056*, *male 27 years)*

Some YPHIV described just simple boredom during adolescent life that prevented them from having good adherence as they used to do.

"*I stopped taking it*. *I did not know why*. *It was just… I did not feel like taking those pills anymore*. *It was hard to explain*. *Even now*, *I do not understand my mood at that time*.*" (057*, *male 28 years)*

Several male YPHIV shared his feeling of being strong and invincible in their adolescence. He believed that he did not need medication to stay healthy. Another guy claimed he was well, even when he did not take his medication on time; his blood test revealed good virologic control.

"*I stopped taking my med for a while*. *I felt strong and as healthy as I used to*. *However*, *my blood test revealed bad results*, *but I was still feeling good with myself*. *(050*, *male 25 years)*

“*I took my med late for a while*, *sometimes I just…took it before or after the planned time*. *Anyway*, *I felt that I was healthy*, *my blood test was good*.*” (059*, *male 28 years)*

### Barriers at the societal level

#### Social activities

Some YPHIV stated that their leisure activities with friends were a primary reason for missing doses. They described that when it occurred again and again and finally became a routine, they did not resume their regular dosing time and failed to adhere to ART.

"*When hanging out with friends*, *I sometimes forgot to bring my med*. *I heard my phone rang in my pocket*, *but I did not want to leave and head back home" (044*, *male 28 years)*

#### Unexpected life situation

In the transitional period, unplanned or unexpected events affected ART adherence. A guy shared his experience escaping from the temple where he resided with monks without bringing the hospital appointment card. He stopped taking the drug when he ran out of it and could not figure out how to get back into the clinic to refill his medication.

“*There was no problem with the drug*. *I was ok with it*, *and no side effect at all*. *The thing was…I escaped from the temple where I lived*. *I did not have the appointment card with me when I ran out of drug*. *I was just…quit it*.*” (052*, *male 22 years)*

Another YPHIV shared about when she worked a shift at a 24-hour convenience store. She was confused with the time of the day, which affected her ART adherence.

“*I started working as a shift*, *switched between day and night shifts*. *I was confused with the time of the day*. *I missed several doses day after day*. *I was so tired*, *sometimes I worked continuously for 7 days without a day-off*.*” (043*, *female 21 years)*

#### Fear of HIV disclosure

Many YPHIV did not disclose their HIV status to anyone outside the family. They described the need to hide their pills and the fear of picking them up when the time came. A man shared that he started his job as a security guard after finishing high school. He always hung out with friends in the evenings and missed his evening dose.

“*I hid my med in the pocket when hanging out with friends*. *I took it quickly when no one saw me*. *I did not want them to see and tease me*. *My friends were crazy*, *and they liked teasing one another about things*.*" (053*, *male 21 years)*

A woman did not disclose to her boyfriend, even after they moved to stay together. She indicated being insecure with life and not knowing what to expect if her boyfriend knew about HIV.

"*It was when I had a new boyfriend that I missed so many doses*. *I was afraid that he would know my status*. *He was a bad-tempered guy and always hurt me*.*" (042*, *female 26 years)*

### Barriers at the healthcare system level

#### Extra payment/medical care cost

The only cause of non-adherence identified by YPHIV in this study was the medical care cost. A young woman stated that she once missed her medication because her father could not afford the drug cost.

“*I missed my med for a while*, *as my dad did not have money to pay for my med*. *He had to pay cash and get reimbursement from his workplace [civil servants’ medical expense] later” (054*, *female 21 years)*

### Facilitators at the personal level

#### Perceived health deterioration

With signs/symptoms of diseases and sickness following virologic failure, YPHIV perceived their health deterioration. The suffering from illnesses reminded them of the need to take medication correctly. They learned the consequences of non-adherence and tried to incorporate ART into their daily life to avoid them.

“*I felt sick at that time*. *It was like having fever off and on*. *I perceived it might relate to frequent missing doses*, *so I tried to take my pills on time*. *I knew it myself even before the doctor talked to me about my virus*.*" (051*, *female 26 years)*

"*I could not walk for a year*. *I was suddenly limb and fell*. *Doctors did not know why*. *Many tests done and at last they said it might be due to me not taking my med well enough*. *I tried to improve myself since then*, *it took a long time until I could walk normally and get a job" (046*, *female 23 years)*

#### Being afraid of hospitalization and medical procedures

Because of the discouraging environment and medical procedures that threatened them during their hospitalization in adolescents’ lives, YPHIV tried to improve their ART adherence to stay away from that suffering.

"*I had been in the hospital*, *an ICU*. *I felt very lonely and suffered from drug infusion into my arm*. *I was extremely painful*. *I am afraid of being like that again" (042*, *female)*

#### Wish to be healthy and move on

In late adolescence, they wanted to have a bright future like their peers, survive in the real world, and earn sufficient money for life. After getting a full-time job, a man described his life perspective as hopeful and ambitious. He perceived that adhering to ART was critical as it would allow him to move toward his future.

" *As a young adult*, *I have a lot of dreams and strong ambition to do things*. *Med is so important to control the virus so that it could not destroy our body and our life*. *We need to be healthy to move forward*.*" (057*, *male 28 years)*

### Facilitators at the societal level

#### Perceived family support

Family is an essential component of life for most people, including YPHIV. Some of them still lived with their parents while having their children. YPHIV stated that they lived and worked with the hope of seeing their future. A female informant mentioned her mother’s words, which she always recalled when the dosing time came. Although she was a grown-up and had children, she could perceive her mother’s love and care for her to be healthy.

"*My mom always told me to take my pills regularly*. *She said I needed to survive for my kids*, *so I did*. *Now they are 10 and 11; both are not aware of my HIV*.*" (060*, *female 28 years)*

#### Desire to complete family without HIV transmission

Most YPHIV learned about HIV transmission and how to prevent HIV from spreading to others since they were school age. Nowadays, with effective treatment and advanced knowledge, PLHIV can have sex, become pregnant, or make others pregnant without the need for artificial fertilization. A male claimed that he started taking his medication correctly as he would like to create his own family and have children. Several young women claimed they resumed good adherence when they became pregnant, as they wanted to protect their babies.

“I *watched the news and saw information about advanced HIV drug development*. *They said that it was possible for people with HIV to have children*. *I meant in a natural way*, *without the need to make a test-tube baby*. *Then I start thinking that…I would better be adherent to my med” (056*, *male 27 years)*

“*I stopped my med for a few years after quitting school*. *Then I became pregnant*, *and the doctor gave me the same med I used to take*. *I took it every day without missing dose until my child was born*.*" (042*, *female 26 years)*

#### Perceived responsibility for their children

YPHIV claimed that their perspective changed in the late adolescent years when they had their child. A woman recalled when she needed to be in the hospital due to an opportunistic infection. While staying away from her children, she realized how important they were. It made her a solid wish to stay healthy and spend the most time with her toddlers at home.

"*I want to be with my kids*. *I could not do that if I was sick*, *unhealthy*, *or had to stay in the hospital*. *I want to see them growing up" (060*, *female 28 years)*

Another woman talked about her strong desire to be alive with her children. It was why she took ART on time and resumed adherence.

"I *was desperate that I did not take my med and felt unwell*. *I thought about killing myself*, *but I did not do*. *I love my kids and I need to be alive for them*.*" (042*, *female 26 years)*

Male participants perceived their family as a primary reason for adherent to ART. A guy with adverse childhood events has struggled after his parents separated. He worked right after finishing primary school as a labourer in several factories. He had a few years without a secure family with non-adherence and virologic failure. Meeting with a supportive partner in his late adolescent years, he changed his idea about life.

"*When I had a wife*, *she supported me to take my med on time*. *We had a child*. *I did not want to die with AIDS*. *I wanted to see my daughter growing up*, *see her go to school*. *I loved them so much*. *That was why I took my med*.*" (049*, *male 25 years)*

### Facilitators at the healthcare system level

#### Service behaviours of healthcare providers

YPHIV mentioned their positive feeling and willingness to maintain ART adherence when meeting with providers who seemed to care about them. Even though doctors prescribed the same medication, different practice styles affected their feelings, perceptions, and ART adherence.

"*The previous doc did not ask me any questions*. *The new [doctor] was much better*. *He cared about me*. *He knew that I was depressed and gave me some med to make me feel better*. *He cheered me up and advised me how to take that big bitter pill*.*" (042*, *female 26 years)*

Meanwhile, some service behaviors might affect adolescents’ treatment adherence without intention. Another female claimed that she resumed good adherence, as she deserved more attention from the doctor.

"The *doctor did not talk to me much*, *when he saw my blood virus [high]*. *He just said that it did not change and sent me out to the pharmacy within a few minutes*. *I thought that that he might talk more if my blood test was good*, *so I tried to take my med regularly*.*" (046*, *female 23 years)*

## Discussion

From interviews with YPHIV, most mentioned barriers and facilitators to ART adherence at the personal level. Work/study-related conditions and individual activities they had to participate in various social environments or life situations inhibited them from adhering to ART as they did at a younger age. At the societal level, HIV-related stigma had a significant role in shaping their adherence behaviors. Meanwhile, the strong facilitators included the determination to stay healthy and move on to the future at the personal level and perceived social support at the societal level.

Our findings indicated that many YPHIV participants could not manage their medication. This supported the low adherence self-efficacy reported in previous study among YPHIV at the median age of 320.2 years [20]. At certain biological ages, caregivers and healthcare providers might expect that YPHIV could make their plan and cope with changing situations. In fact, cognitive impairment associated with HIV infection might be an underlying cause of functional impairment [21]. The inability to manage medication and fear of disclosure was similarly described as a part of youth’s experiences regarding ART adherence in a study conducted among Thai YPHIV aged 14–21 years who received ART in community hospitals in 2010–2011 [22]. When youth get a job, they would instead devote themselves to it and prioritize their job responsibility over the need to take medication. Healthcare providers could have a role in guiding and coaching them to make individual plans for managing their medication in different situations. YPHIV should be empowered to maintain their daily discipline to minimize missing doses while not interrupting their day-to-day routine.

Regimen fatigue and resistance to taking medication were reported in older children to adolescent years, as previously mentioned in the U.S. study [23]. We supported that these challenges could persist until late adolescence and young adulthood, as some YPHIV in our study reported being discouraged, feeling hopeless, and having depressive symptoms during adolescence. The findings of desperation about their future while believing they were invincible and invulnerable to illnesses were noted. Either way, they perceived no need to take medication. The desperation was in line with the previous Thai youth study, which reported boredom and discouragement because of the long treatment duration for an incurable disease [22]. On the contrary, the feelings of being invincible and invulnerable to illnesses were in contrast with a U.S. study among youth with HIV aged between 17–25 years, where only a few expressed a feeling of invulnerability to the consequences of HIV in the focus group discussion [24]. Our findings might reflect inadequate health literacy or misunderstanding about the natural history of HIV infection as we found some YPHIV stopped taking their medication due to side effects while believing that they could stay healthy without ART. This misbelief indicated the need for more HIV education, including how drugs control viral replication and how the immune system works. For adverse effects of ART, YPHIV should be encouraged to advocate for themselves and negotiate with their providers to get a more tolerable regimen that does not interfere with their daily activities. The meta-analysis suggested the effects of pharmacist care on improving ART adherence and HIV treatment outcomes [25]. More pharmacist involvement in HIV multidisciplinary care team should be encouraged. Interventions to provide updated HIV knowledge and health education to YPHIV during the transitional period might be considered by institutions providing HIV services, i.e., support groups or mHealth. The Tanzanian study reported the benefit of using peer support groups on improving health outcomes, HIV education, and psychosocial in youth with HIV [26]. Besides face-to-face education sessions, mHealth could be another approach to enhance HIV education, facilitate two-way communication between providers and YPHIV, and promote ART adherence [27].

The most frequent barrier at the societal level was perceived HIV-related stigma. We learned that most study participants did not socially disclose their HIV status. In most circumstances, YPHIV would avoid taking medication in the presence of other people as they did not want to explain what the medicine was for. This challenge was in line with the report from a U.S. study on adults with HIV, in which participants claimed that taking pills every day was associated with a greater chance of revealing their HIV status to others [28]. A study among adolescents with perinatal HIV in Botswana reported that fear of stigma influenced the choice between taking medication at the dosing time and other daily activities [29]. According the a previous study among Thai adults living with HIV in upcountry areas, HIV-related stigma was identified as a primary concern where misconceptions surrounding risk factors for transmission and lack of up-to-date knowledge on modern HIV treatment and prevention were presented in communities [30]. Stigma impacted their adherence behaviors and mental health while living in close-knit communities. Social support is associated with better quality of life in people living with HIV [31]. However, YPHIV with asymptomatic HIV might not feel like getting more support than others without HIV. They only did not want to be different from their peers or siblings. Healthcare providers could have a role in creating a less stigmatized environment, providing updated information, and guiding YPHIV on how to talk to others, if necessary, about disease and medication. Cultural appropriate intervention to reduce stigma in the community that involves people living with HIV in designing and implementation is also required [32]. Stigma-reduction interventions for YPHIV might include cognitive behavioral therapy to boost self-esteem and improve stress management, which was reported from studies in low-middle-income countries as an effective approach [33, 34].

The facilitators to ART adherence included perceiving their health deterioration. After being non-adherent, some YPHIV felt unhealthy and realized it was related to their frequent missing dose or stopped taking medication. Some had revisiting opportunistic infections during virologic failure. They mentioned their being afraid of hospitalization and medical procedure-related sufferance. The fear was similar to a report from Botswana, in which adolescents reported their fear of getting sick and dying as a significant motivator for adherence [29]. YPHIV realized from their negative experiences that being adherent to ART was the way toward ordinary life as that of others without HIV.

Nevertheless, we did not think that everyone needs to have direct experience. YPHIV could learn from other people. Hence, peer support groups or social networks where they could share lessons and support one another might benefit YPHIV, perhaps through a virtual platform or internet-based support intervention, which reported promising outcomes [35, 36]. Many young people stated they wish to be healthy, live longer, and move toward a better future. This idea emerged after episode (s) of suboptimal adherence, usually in late adolescents. Thus, it was like the previous study in Thai adults with HIV [30], in which participants’ determination to stay healthy was a key component of good adherence.

The role of healthcare providers remained critical as YPHIV had a long-term relationship with HIV services since childhood; they deserved attention and reassuring words when they came to the clinic. The Indonesian study documented the role of relationships between healthcare providers and PLHIV, as well as their attitudes as enabling factors for PLHIV access to services [37]. We found different situation as in Thailand ART is free of charge under several healthcare schemes and is easy to access in government or private hospitals. There were some logistic problems related to HIV clinic visits that could be fixed by the healthcare team, who are aware of and understand the situation. Individual counseling and problem solving on a case-to-case basis focusing on their concern is necessary and would benefit YPHIV with different needs. We proposed additional training for providers to deliver youth-friendly services in the existing HIV clinics. In addition, healthcare providers could also bring in and work with non-government organizations with youth-focusing missions to support YPHIV in other dimensions of life.

The strength of this study is that we obtained information from young adult survivors. They were born during the HIV epidemic in northern Thailand and have passed through a long journey of life. The study had several limitations. We included YPHIV, who could resume virologic suppression after failure. Their barriers to adherence might not be as severe or difficult to manage as those of other YPHIV who could not resume virologic suppression. We recruited YPHIV who were willing to share their experiences. They might have different characteristics, attitudes, and more reasons for non-adherence than those who denied sharing. Our study population did not include unreachable YPHIV who are at a higher risk of treatment failure, loss-to-follow-up, morbidities, and mortality. We might need to make more effort to reach them or get help from community-based organizations to reach them. Further research to address their needs is warranted to understand the pitfalls in the current HIV service system and allow us to design and implement interventions for YPHIV to prevent untoward outcomes.

## Conclusions

The main factors affecting ART adherence in adolescents are personal. We documented the fear of HIV disclosure associated with non-adherence at the societal level. The determination to be healthy and have a promising future were key facilitators to adherence. Our findings reinforce the importance of establishing youth-friendly services in the existing HIV care setting. Youth providers should allocate more time for tailored individual counselling to maximize their adherence self-efficacy during the transitional period of YPHIV. Using other novel approaches like mHealth, online media, and involvement of social support from different sectors, while being aware of unintended HIV disclosure, might be beneficial to maximize adherence self-efficacy during the transitional period of YPHIV.
